# Health state prediction with reinforcement learning for predictive maintenance

**DOI:** 10.3389/frai.2025.1720140

**Published:** 2026-01-12

**Authors:** Anastasis Aglogallos, Alexandros Bousdekis, Stefanos Kontos, Gregoris Mentzas

**Affiliations:** Information Management Unit (IMU), Institute of Communication and Computer Systems (ICCS), School of Electrical and Computer Engineering, National Technical University of Athens (NTUA), Athens, Greece

**Keywords:** deep learning, degradation prediction, Industry 4.0, machine learning, predictive maintenance, reinforcement learning

## Abstract

**Introduction:**

Predictive maintenance has emerged as a critical strategy in modern manufacturing, in the frame of Industry 4.0, enabling proactive intervention before equipment failure. However, traditional machine learning approaches require extensive labeled data and lack adaptability to evolving operational conditions. On the other hand, Reinforcement Learning (RL) enables agents to learn optimal policies through interaction with the environment, eliminating the need for labeled datasets and naturally capturing the sequential, uncertain dynamics of equipment degradation.

**Methods:**

In this paper, we propose an approach that incorporates four model-free RL algorithms, namely Proximal Policy Optimization (PPO), Advantage Actor-Critic (A2C), Deep Deterministic Policy Gradient (DDPG), and Soft Actor-Critic (SAC). We formulate the problem as a Markov Decision Process (MDP), which is solved with the aforementioned RL algorithms.

**Results:**

The proposed approach is validated in the context of CNC machine tool wear prediction, using sensor data from the 2010 PHM Society Data Challenge. We examine algorithmic performance across four custom made environments, corrective and non-corrective environments both with and without delay correction mechanisms in order to compare learning dynamics, convergence behavior, and generalization aspects. Our results reveal that PPO and SAC achieve the most stable and efficient performance, with SAC excelling in structured environments and PPO demonstrating robust generalization. A2C shows consistent long-term learning, while DDPG underperforms due to insufficient exploration.

**Discussion:**

The findings highlight the potential of RL for predictive maintenance applications and underscore the importance of aligning algorithm design with environment characteristics and reward structures.

## 1 Introduction

In the era of Industry 4.0, predictive maintenance has become a foundational component of intelligent manufacturing, aiming to reduce unexpected downtime, optimize maintenance scheduling, and extend equipment life through data-driven insights (Aivaliotis et al., 2019; Fordal et al., 2023). The integration of high-frequency sensors and advanced data acquisition systems has enabled real-time condition monitoring, but the inherent complexity and variability of industrial environments continue to challenge traditional Machine Learning (ML) techniques (Turner et al., 2019; Lepenioti et al., 2020). These models often rely on large volumes of labeled failure data, assume stationary behavior, and struggle to generalize across diverse operational contexts (Bousdekis et al., 2021).

Reinforcement learning (RL) offers a compelling alternative, as it enables agents to learn optimal policies through interaction with the environment, eliminating the need for labeled datasets and naturally capturing the sequential, uncertain dynamics of equipment degradation (Siraskar et al., 2023). Recent studies have demonstrated the viability of RL in predictive maintenance tasks such as fault detection, health index estimation, and direct maintenance scheduling (Siraskar et al., 2023; Feng and Li, 2022; Ong et al., 2022). However, real-world applications remain limited, and comprehensive evaluations of RL algorithms under realistic conditions are still scarce (Siraskar et al., 2023).

In this paper, we propose an approach that incorporates four model-free RL algorithms, namely Proximal Policy Optimization (PPO), Advantage Actor-Critic (A2C), Deep Deterministic Policy Gradient (DDPG), and Soft Actor-Critic (SAC). To do this, we formulate the problem as a Markov Decision Process (MDP), which is solved with the aforementioned RL algorithms. The proposed approach is validated in the context of CNC machine tool wear prediction, using sensor data from the 2010 PHM Society Data Challenge. We examine algorithmic performance across corrective and non-corrective environments, both with and without delay correction mechanisms in order to compare learning dynamics, convergence behavior, and generalization aspects.

The rest of the paper is organized as follows. Section 2 presents the literature review on the use of RL in predictive maintenance. Section 3 describes our proposed approach for health state prediction with RL. Section 4 presents the application of the proposed approach to a CNC milling machine and the experimental results. Section 5 concludes the paper and outlines our plans for future work.

## 2 Literature review

Predictive maintenance has become a pillar of Industry 4.0, with the goal of minimizing unplanned downtime, optimizing maintenance schedules, and extending equipment life by forecasting degradation and impending failures (Aivaliotis et al., 2019; Bousdekis et al., 2019). The sensory technologies support real-time condition monitoring using continuous sensor signals such as vibration, acoustic emissions, force, and temperature (Huang et al., 2017; Pech et al., 2021).

The first predictive maintenance systems were mainly rule-based and deterministic, limiting their effectiveness in dynamic and nonlinear industrial environments (Bousdekis et al., 2018). The introduction of ML represented a major advancement, enabling the modeling of complex degradation patterns (Carvalho et al., 2019). Supervised ML techniques, such as decision trees (Frangopol et al., 1997), Support Vector Machines (SVM) (Ding et al., 2008; Susto et al., 2013), random forests (Kabir et al., 2018), gradient-boosted trees (e.g., XGBoost) (Ma et al., 2020), and Bayesian Networks (Yang et al., 2023a; Yang et al., 2023b) have been widely used for fault classification and prognostics (Dalzochio et al., 2020). Other techniques such as Principal Component Analysis (PCA) (Eke et al., 2017) and regression-based ML models (Susto et al., 2014) have also contributed to predictive maintenance by reducing dimensionality and supporting flexible diagnostic systems (Sun et al., 2007). Deep learning has further improved predictive maintenance performance by enabling models to learn directly from raw time-series data. Long Short-Term Memory (LSTM) networks (Zheng et al., 2017; Sayyad et al., 2022) and Convolutional Neural Networks (CNNs) (Sateesh Babu et al., 2016) have been effectively applied to degradation modeling and regression tasks (Serradilla et al., 2022).

However, these approaches and algorithms face notable limitations. They typically require large amounts of labeled data, particularly historical failure records, which are often unavailable or expensive to collect (Shakya et al., 2023). Moreover, they assume stationary input–output mappings, making their application to real-world settings challenging, because the equipment behavior evolves over time (Dalzochio et al., 2020). Retraining models to adapt to new machines or operational conditions is time-consuming and often suffers from concept drift (Lehmann et al., 2020). In this realm, RL has emerged as a promising alternative to traditional ML for predictive maintenance (Ogunfowora and Najjaran, 2023). Unlike supervised learning, RL does not depend on labeled data; instead, it learns optimal decision policies through interaction with the environment (Shakya et al., 2023). In predictive maintenance applications, RL agents monitor system states (e.g., sensor measurements or health indicators), take actions such as predicting wear or scheduling maintenance, and receive rewards based on objectives like minimizing repair costs or downtime (Siraskar et al., 2023).

This trial-and-error learning paradigm naturally captures the sequential and uncertain characteristics of maintenance decision-making, where current actions influence future system outcomes (Lewis et al., 2012; Sutton and Barto, 1998). Siraskar et al. (2023) have reviewed RL applications across tasks such as early fault detection, health index estimation, and direct maintenance decision support. Ogunfowora and Najjaran (2023) further highlight RL’s capacity to integrate short-term equipment states with long-term maintenance cost considerations, something traditional deep learning models struggle with under shifting conditions. However, these works typically focus on single algorithms or single-task environments. In contrast, our study contributes a multi-environment, multi-algorithm experimental framework that reveals how RL performance depends on problem formulation, reward structure, and environment corrective behavior.

Several RL algorithms have been applied to the predictive maintenance domain. Value-based methods, such as Q-learning and Deep Q-Networks (DQN), are well-suited for discrete action spaces. According to (Ogunfowora and Najjaran, 2023), more than 70% of reviewed studies utilized Q-learning variants. For instance, Latifi et al. (2021) employed DQN and PPO for infrastructure asset management, effectively balancing cost and performance. Some works have shifted towards policy-gradient and actor-critic methods for their effectiveness in continuous state-action spaces (Khadka and Tumer, 2018). Algorithms such as PPO, A2C, DDPG, and SAC combine value estimation with policy learning for enhanced decision control. Wang et al. (2024) demonstrated that a CNN–BiLSTM-enhanced DDPG model outperformed conventional methods in bearing RUL prediction. SAC, notable for its entropy-based exploration, has shown effectiveness in high-stakes applications like aircraft engine maintenance, where sample efficiency is critical. These findings suggest that while model-free methods like Q-learning and DQN perform well in discrete domains, actor-critic algorithms offer greater control precision and adaptability in complex industrial scenarios. Recent studies have also explored advanced RL-based frameworks for predictive maintenance. Abbas et al. (2024) propose a hierarchical approach that combines an Input–Output Hidden Markov Model with DRL to improve interpretability and sample efficiency in safety-critical applications. Zhao et al. (2024) introduce TranDRL, which integrates Transformer-based RUL prediction with DRL-driven maintenance recommendations and human-in-the-loop feedback. In the machining domain, Kaliyannan et al. (2024) evaluate RL algorithms for tool-condition monitoring and show that SARSA outperforms both deep learning and other RL baselines for classifying tool wear from vibration signals. Furthermore, literature reviews by Ogunfowora and Najjaran (2023) and Siraskar et al. (2023) support the conclusion that well-calibrated deep RL models can exceed traditional ML baselines in simulated environments.

Despite their potential, many RL-based predictive maintenance approaches remain at the proof-of-concept stage, often relying on synthetic data or simplified simulation environments (Siraskar et al., 2023). The lack of real-world benchmarks and standardized evaluation protocols limits cross-study comparability. Moreover, safe deployment in industrial settings is constrained by the need for robust, cautious exploration mechanisms, particularly in high-risk systems where untested policies could cause costly failures (Çınar et al., 2020). These challenges underscore the need for ongoing research focused on developing generalizable, scalable, and real-time RL frameworks that can adapt to evolving operational conditions in industrial predictive maintenance applications.

## 3 The proposed approach for health state prediction with reinforcement learning

This Section describes the proposed approach for health state assessment of manufacturing equipment with RL in order to realize the predictive maintenance paradigm. The proposed approach consists of the following steps: (i) *Data Structuring* (Section 3.1); (ii) *Data Preprocessing* (Section 3.2); *(iii) MDP Modelling* (Section 3.3); *(iv) Solving with RL* (Section 3.4); and, *(v) Evaluation* (Section 3.5).

Initially, data collected from sensors are processed and subsequently transformed into a MDP model. Through the MDP, a suitable environment is developed for the implementation of various RL algorithms, which is trained on the processed dataset. The last step is, after the training, the algorithms will be evaluated to determine their effectiveness. If their performance is satisfactory, they can be utilized to perform predictions about the equipment degradation. The end goal is to develop an RL-based Predictive maintenance agent capable of making autonomous decisions to predict the health state and the wear. Table 1 presents the steps of the proposed approach along with their inputs and outputs throughout the data pipeline. The proposed approach incorporates model-free RL algorithms.

**Table 1:** The steps of the proposed approach along with their inputs and outputs.

Section	Step	Input	Output
3.1	Data Structuring	Sensor Signals	Structured Data
3.2	Data Preprocessing	Structured Sensor Data	Transformed Data
3.3	MDP Modelling	Transformed Data	Observation Space, Action Space, Reward Function, Episode Information
3.4	Solving with RL	Observation, Reward	Action
3.5	Evaluation	Trained RL Model	Evaluation Metrics

Unlike previous studies that examine single RL algorithms or simplified maintenance scenarios, this work introduces a unified and reproducible RL evaluation framework specifically tailored for equipment wear prediction. Our contributions are fourfold: (i) we propose a novel 2 × 2 environment design (corrective/non-corrective × delayed/non-delayed rewards) that enables systematic investigation of how RL agents interpret different degradation formulations; (ii) we integrate domain-informed reward shaping by adapting the PHM competition score as an RL reward function; (iii) we provide the first comprehensive cross-algorithm comparison of PPO, A2C, SAC, and DDPG on the Li, 2021 dataset using a unified pipeline; (iv) we present open-source implementations and reveal empirical insights—such as differential sensitivity to reward delay and overfitting tendencies—that have not been documented in prior RL-based predictive maintenance studies.

### 3.1 Data structuring

In this step, the sensor-generated time-series data is formulated in a table form with each row corresponding to a sensor value at a specific timeframe. These sensors capture critical parameters such as vibrations, force, spin, temperature, pressure over time and other related functional characteristics. The input parameters include not only the raw sensor readings but also derived features that help in capturing the underlying patterns and trends in the data. The output of this step is structured data that provides a better understanding of the use case and can be subsequently processed in an effective manner.

### 3.2 Data preprocessing

Through data preprocessing, it becomes possible to create a more manageable and informative dataset. This step takes as input the structured data and includes the following steps: (a) *data visualization*: Exploratory Data Analysis (EDA) supporting understanding of the data’s characteristics, distributions, anomalies, inconsistencies, and irregularities; (b) *data cleaning*: removing noise and errors to ensure consistency and robustness; (c) *feature extraction*: transforming raw data into meaningful features in order to handle the high-dimensional nature of sensor data. As far as feature extraction is concerned, the proposed approach adopts a set of time-domain features, characteristics derived from analyzing time-series data-data points collected or observed at different time intervals, which have been proved effective in various manufacturing settings, particularly in the presence of vibration sensors (Wilson, 2017; Kumar et al., 2022). The proposed approach incorporates the features presented in Table 2. The output of this step is the transformed data that feeds into the MDP modelling step.

**Table 2:** The time-domain features of the proposed approach.

Feature	Definition	Formula
Mean	It is derived from the average value over a given time period. It provides a sense of central tendency.	$\text{Mean}=\mu =\frac{1}{Ν}\sum_{\iota =1}^{Ν}x_{i}$
Root Mean Square (RMS)	It is a signal processing feature which measures the square root of the mean of the squares of all values in the time series.	$\mathrm{RMS}=\sqrt{\frac{1}{Ν}\sum_{i=1}^{N}{x_{i}}^{2}}$
Crest Factor	It is a signal processing feature and is calculated by the ratio between the peak value and the RMS value, indicating the dynamic range of a signal.	$\text{Crest Factor}=\frac{\max[x_{i}]}{\mathrm{RMS}}$
Average power	It is the average quantity of work done or energy utilised per unit of time.	$\mathrm{avg}.\text{power}=\frac{1}{Ν}\sum_{i=1}^{N}{x_{i}}^{2}$
Skewness	It is a high-order statistical feature which indicates the asymmetry of the data distribution. A skewed time series may suggest trends or biases.	$\text{Skewness}=\frac{E\;[{\left(x_{i}-\underline{x}\right)}^{3}]}{\mathrm{RM}S^{3}}$
Kurtosis	It is a high-order statistical feature which reflects the “tailedness” of the data distribution, which can reveal the presence of outliers or extreme events.	$\text{Kurtosis}=\frac{\frac{1}{N}\sum_{i=1}^{N}{\left(x_{i}-\underline{x}\right)}^{4}}{\mathrm{RM}S^{4}}$

### 3.3 MDP modelling

This step receives the transformed data and creates the MDP model that represents the degradation process of the equipment. In this way, it defines the observation space, the action space, the reward function, and the episode information, that will be subsequently used by the RL algorithms, thus defining the environment in which the agent will take actions. The MDP consists of the following components: States Space, Action Space, Transition Model, Reward Function and the starting state distribution ρ_0_. Therefore, an MDP is a 5-tuple, (S, A, R, P, ρ_0_), where: S is the set of all valid states; A is the set of all valid actions; R:S×A×S→R is the reward function, with $r_{t}=R(s_{t},a_{t},s_{t+1})$ ; P:S×A→P(S) is the transition probability function, with P(s′∣,s∣,a) being the probability of transitioning into state s′ if you start in state s and take action a ; ρ_0_ is the starting state distribution (Garcia and Rachelson, 2013).

Below, we describe in detail the MDP model specifically crafted for equipment Health State Prediction:

- State $S_{t}$ : The state at any time step t is represented by the condition of the machine parts that is represented by the value of the sensors attached to the machines at t. The vector containing all the information of the environment is called the state of the environment. The set S contains all the valid states $S_{t},\forall t$ of the environment, therefore it contains all the different wear states and values the examined equipment may attain.
- Action $a_{t}$ : The action space consists of a n-dimensional space representing the wear of the n components of the equipment, i.e., $a_{t}=[\text{actio}n_{1},\text{actio}n_{2},\ldots ,\text{actio}n_{n}]$ , where: $\text{actio}n_{1}\;(\text{predictio}n_{1})$ : is the prediction of the agent for the additional wear of the component 1 at timestep t, $\text{actio}n_{2}\;(\text{predictio}n_{2})$ is the prediction of the agent for the additional wear of the component 2 at timestep t, and $\text{actio}n_{n}\;(\text{predictio}n_{n})$ is the prediction of the agent for the additional wear of the component n at timestep t. The action is the corresponding additional wear that the agent predicts for the component. This vector can be either discrete or continuous; however, a continuous action space is more fitting, since the wear is not a discrete metric.
- Transition Probabilities (P): The transition probabilities define how the state changes in response to actions. Depending on how the machine operates, the resulting induced wear is a stochastic phenomenon. Therefore, the way predictions are managed is through calculating the probability of the given wear occurring given the current state. Due to the Markov property, the transitions only depend on the most recent state and action, and not on prior history. The probability of transitioning to state $s_{t+1}$ given the current state $s_{t}$ and action $a_{t}$ is denoted by $P(s_{t+1}|,s_{t}|,a_{t})$ .
- Reward $R_{t}$ : The reward function is designed to provide feedback to the agent based on the accuracy of its predictions. Depending on the objective, a different reward function will be used, penalizing bad behavior and rewarding good behavior-actions. The reward can be computed based on the difference between the predicted wear and the actual wear observed. Although this is a credible method, more advanced ways of scoring the reward are usually used, called score functions. The score functions behave differently in the case of a bad action and a good action in order to help the agent learn more effectively. The way a score function evaluates actions determines what and how the agent learns. Typically, defining the score function is a challenging step that requires a deep understanding of the problem we aim to solve. The general formula of a reward is: $R_{t}=\text{Scor}e_{f}(\text{predicted value}-\text{actual value})$ .
- Starting state distribution ρ_0_ defines the probability distribution over the initial states from which the agent begins. This distribution is crucial as it sets the initial conditions and significantly influences the early stages of learning and exploration. Therefore, ρ_0_ represents the likelihood of various initial wear conditions and sensor readings of the machine at the start of the monitoring period. The initial state could be determined based on historical data, reflecting common starting conditions observed in past machine operations. A well-defined ρ_0_ ensures that the agent experiences a realistic range of initial conditions during training, promoting robust learning and better generalization to real-world scenarios. It is bad practice to always have the same initial condition since it does not reflect real life conditions.
- Trajectories / Episodes: A trajectory *τ* consists of a sequence of states and actions: $\tau =(s_{0},a_{0},s_{1},\alpha_{1},\ldots )$ . The initial state s_0_ is randomly drawn from the start-state distribution, often represented by ρ_0_: $s_{0}∼\rho_{0}(\cdot )$ . State transitions, which describe changes in the world from state $s_{t}$ at time t to state $s_{t+1}$ at time t+1 , are influenced by the most recent action $a_{t}$ . Trajectories are often referred to as episodes or rollouts.

The MDP model results in a solution specifying the agent’s actions for any state it may encounter, i.e., a policy. The policy is denoted by π , and π(s) is the action that policy π recommends for state s . Regardless of the outcome of the action, the resulting state will belong to the policy, and the agent will know what to do next. The quality of a policy is measured in terms of the expected utility of the possible histories of the environment generated by the policy. An optimal policy is a policy that gives the highest expected utility. The utility function allows the agent to choose actions using the maximum expected utility principle, to choose the action that maximizes the reward for the next step plus the expected utility of the subsequent state of the environment. The output observation space, action space, reward function, and episode information that are derived from the MDP model feed into the subsequent step to be solved with the use of RL algorithms.

### 3.4 Solving with RL

The MDP model is solved with RL, which selects a policy that maximizes the expected return when the agent acts according to it. Therefore, RL consists of the agent and the environment. The environment represents the world in which the agent resides and with which it interacts. During each interaction step, the agent observes a (potentially partial) view of the world’s state and then decides on an action to take. The environment changes in response to the agent’s actions but can also change independently. The agent receives a reward signal from the environment, which measures the value of the current state. The agent’s objective is to maximize its cumulative reward, known as the return.

Our proposed approach incorporates PPO, A2C, DDPG, and SAC, These algorithms are categorized into off-policy and on-policy methods, as shown in Table 3. An off-policy learner learns the value of the optimal policy independently of the agent’s actions, while an on-policy learner learns the value of the policy being carried out by the agent including the exploration steps (Gu et al., 2017). The algorithms are described below.

**Table 3:** The classification of the implemented RL algorithms to learning policies.

Learning policy	RL Algorithms
On-policy	PPO
	A2C
Off-policy	DDPG
	SAC

#### 3.4.1 PPO

The PPO Algorithm (Schulman et al., 2017) is designed to take the largest possible improvement step on a policy using the available data without risking performance collapse. The main idea is to keep the new policy not too far from the old policy after an update, using clipping to prevent large updates. PPO is an on-policy algorithm suitable for environments with both discrete and continuous action spaces. The PPO algorithm trains a stochastic policy in an on-policy method, meaning it explores by sampling actions based on the current stochastic policy. The randomness in action selection depends on initial conditions and the training process. Over time, the policy becomes less random as the update rule encourages exploiting previously discovered rewards. This reduction in randomness can lead to the policy within a local minimum. PPO was selected for this task due to its stability and reliable performance in continuous control tasks. As an on-policy algorithm, its core design, which uses a clipped objective function, prevents large, destabilizing policy updates. This characteristic is highly desirable for a predictive maintenance problem, where erratic predictions or policy collapse would be detrimental.

#### 3.4.2 A2C

The A2C Algorithm (Mnih et al., 2016) is a synchronous RL algorithm that enhances the Asynchronous Advantage Actor-Critic (A3C) by synchronizing data collection across multiple parallel workers. This approach mitigates the high variance and noisy gradients seen in vanilla policy gradients by incorporating a baseline, typically the value function, which stabilizes learning. In A2C, the Actor updates the policy distribution based on feedback from the Critic, who estimates the value function. The advantage function A(s,a)=Q(s,a)−V(s) quantifies how much better taking action a in state s is compared to the average action. This advantage function helps calculate the policy gradient, ensuring policy parameters are updated to maximize expected returns. The synchronous nature of A2C ensures efficient and stable training, suitable for various complex environments. This method effectively balances exploring new actions with exploiting known rewards, leading to robust and efficient policy learning. A2C was chosen for this evaluation as it represents a synchronous and more stable implementation of the foundational actor-critic framework.

#### 3.4.3 DDPG

The DDPG (Silver et al., 2014; Lillicrap et al., 2016) is an algorithm that simultaneously learns a Q-function and a policy. Using off-policy data and the Bellman equation, it learns the Q-function, and this Q-function is then used to optimize the policy. DDPG is closely related to Q-learning, sharing the motivation that if the optimal action-value function $Q^{*}(s,a)$ is known, the optimal action $a^{*}(s)$ in any given state can be found by solving: $a^{*}(s)=\text{argma}x_{a}Q^{*}(s,a)$ . In DDPG, a deterministic policy is trained in an off-policy manner. Due to its deterministic nature, the policy might not initially explore a sufficient range of actions to gather valuable learning signals if explored on-policy. To enhance exploration, noise is added to the actions during training. To improve the quality of training data, the noise scale can be gradually reduced as training progresses. During testing, no noise is added to the actions, allowing the policy to fully exploit what it has learned. As a foundational off-policy algorithm for continuous control, DDPG was selected to serve as a critical baseline in this paper. The inclusion of DDPG enables the comparison of the effectiveness of a deterministic policy gradient approach against the stochastic policy methods (PPO and SAC) and the synchronous actor-critic (A2C) in the context of the health state prediction problem.

#### 3.4.4 SAC

The SAC Algorithm (Haarnoja et al., 2018a; Haarnoja et al., 2018b) optimizes a stochastic policy using an off-policy approach, creating a bridge between stochastic policy optimization and DDPG-style methods. The stochastic nature of SAC’s policy benefits from an effect similar to target policy smoothing. Entropy regularization is a key feature. The policy is trained to balance expected return with entropy, which measures the randomness in the policy. This balance is closely related to the exploration-exploitation trade-off: higher entropy promotes more exploration, potentially accelerating learning in later stages. Additionally, it helps prevent the policy from prematurely converging to suboptimal solutions. SAC was included in algorithm stack as it represents a state-of-the-art off-policy algorithm for continuous action spaces. Its key feature, maximum entropy regularization, fundamentally changes the objective by encouraging the agent to explore as widely and randomly as possible while still maximizing rewards. SAC was chosen in order to investigate whether this advanced, built-in exploration mechanism would enable the agent to discover more effective and sample-efficient policies for health state prediction compared to the other methods.

### 3.5 Evaluation

This step evaluates and compares the trained RL models by computing various evaluation metrics. The RL optimization problem is solved within a custom environment which simulates a system where an agent makes predictions about the wear on certain components in a manufacturing process. To evaluate the RL algorithms in each environment, we use the following metrics:

- Mean Episode Length (Bojun, 2020). It indicates the average duration of an episode before a terminal state is reached. In the context of predictive maintenance, an episode could represent the operational period of a machine before a maintenance intervention is required. A longer mean episode length could suggest that the learned policy is effective at preventing failures and prolonging the operational time of the equipment.
- Mean Episode Reward (Ladosz et al., 2022). It represents the average cumulative reward obtained per episode. In the context of predictive maintenance, the reward function can be designed to encapsulate various factors such as operational efficiency, maintenance costs, downtime, and the occurrence of failures. A higher mean episode reward indicates that the policy is effective in balancing the trade-offs between these factors.

## 4 Implementation and results

### 4.1 Technical implementation

The implementation utilizes a technology stack centered around Python, using various libraries and frameworks to perform data manipulation and RL. The primary helper libraries used for general tasks include NumPy for numerical computations and array handling, Pandas for data manipulation and analysis of table data, and Matplotlib for data visualization. Stable Baselines 3 is employed for RL. Stable Baselines 3 is a library based on OpenAI Baselines. It is built on top of the PyTorch framework and offers a big arsenal of tools designed to help with the development and deployment of RL models. The library features an API that simplifies the setup, training, and evaluation of RL models. In addition, Stable Baselines 3 offers a range of utilities that streamline the RL development process. These include tools for environment checking, vectorized environment handling, policy evaluation, and results plotting. To support file system operations, the project uses the os library for interacting with the operating system and Pathlib for handling file paths for object-oriented purposes. The time library is also included to manage time-related functions and measure execution time.

**Figure 1:**
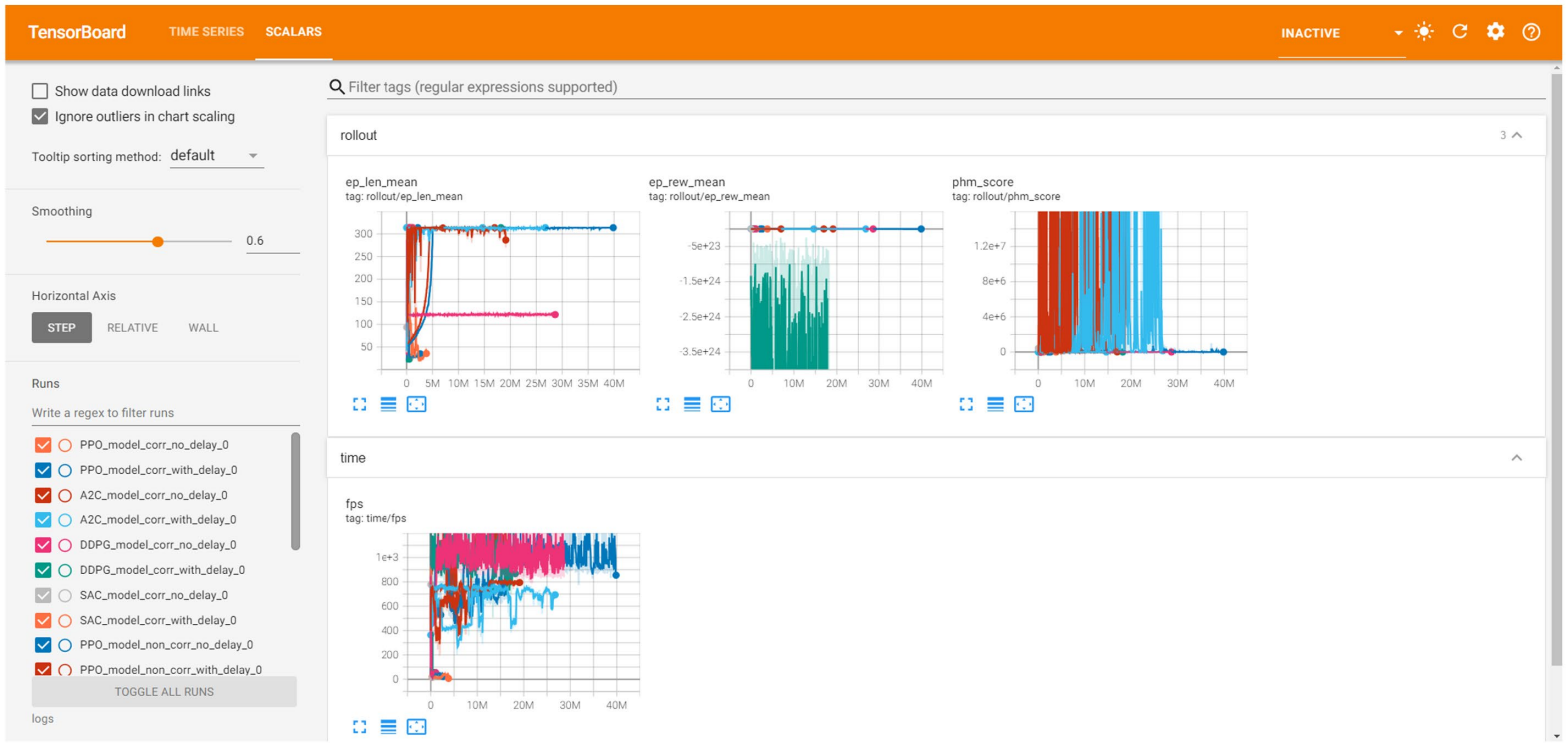
Scalars tab TensorBoard UI.

TensorBoard was used for the real-time monitoring and visualization of model training metrics. Tensorboard is a visualization toolkit included with TensorFlow and is designed to help users understand and debug ML models. It allows the visualization of various training metrics and provides insights into model performance helping in the optimization process. TensorBoard operates through a logging process during model training. The TensorBoard user interface comprises several dashboards and tools for the detailed monitoring and analysis of model training. The Scalars Dashboard (Figure 1) displays plots of metrics such as average reward and average episode length over time, which is useful for tracking the model’s improvement with respect to the number of training iterations. The Time Series tab in TensorBoard allows for a more detailed examination of metrics over time, providing thorough insights into how specific values change throughout the training process.

### 4.2 Application to a CNC milling machine

**Figure 2:**
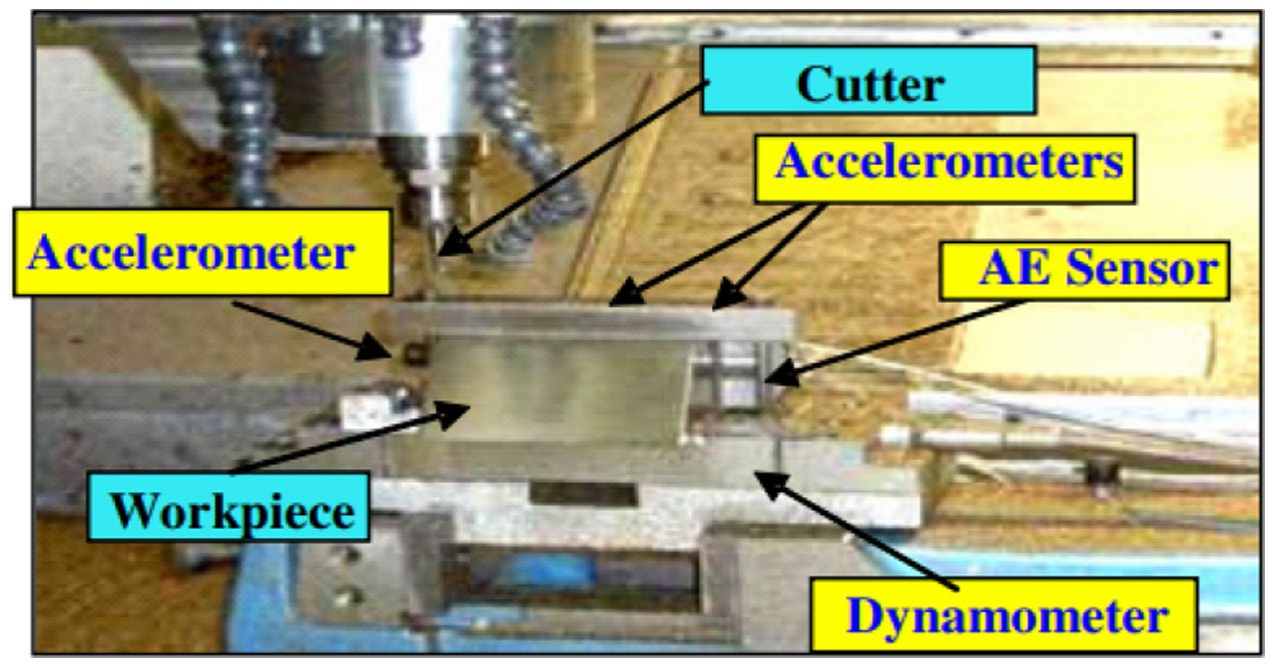
The high-speed CNC milling machine along with its installed sensors (Li, et al., 2009).

The dataset used to train the models is taken from the 2010 PHM Society Conference Data Challenge (Li, 2021). It was derived from a high-speed CNC milling machine, including 6 mm ball nose tungsten carbide cutters, using dynamometer, accelerometer, and acoustic emission sensors (Figure 2). The dataset consists of 6 individual cutter records, c1 to c6. Records c1, c4 and c6 are training data, and records c2, c3, and c5 are test data. Each training record contains one “wear” file that lists wear after each cut in 10^-3 mm, and a folder with 315 individual data acquisition files (one for each cut). The data acquisition files are in .csv format, with seven columns, corresponding to: Force (N) in X dimension, Force (N) in Y dimension, Force (N) in Z dimension, Vibration (g) in X dimension, Vibration (g) in Y dimension, Vibration (g) in Z dimension, AE-RMS (V). The spindle speed of the cutter was 10,400 RPM; feed rate was 1,555 mm/min; Y depth of cut (radial) was 0.125 mm; Z depth of cut (axial) was 0.2 mm. Data was acquired at 50 KHz/channel.

Each cut file in the dataset consists of approximately 250,000 rows, with each row containing 7 data parameters, leading to a highly complex observation space of 1,750,000 dimensions for the agent to work with. To address the curse of dimensionality and extract meaningful features, the focus was on deriving the time-domain features for each input parameter recorded by the sensors. By extracting these 6 features (mean, rms, crest factor, average power, skewness, kurtosis) for each of the 7 sensor parameters (Force in X, Y, Z axis; Vibration in X, Y, Z axis; and AE-RMS), the dimensionality of the data was reduced to 42 features per cut. After extracting the time-domain features, normalization was performed to a range of 0 to 1 to ensure uniformity and improve the performance of the learning algorithms. Normalization was conducted using min-max scaling, which adjusts the values in each feature to fall within the specified range, enhancing the convergence rate of the training process. Then, the wear flute values were included into the dataset, ensuring a complete dataset that includes both the extracted features and the wear measurements.

**Figure 3:**
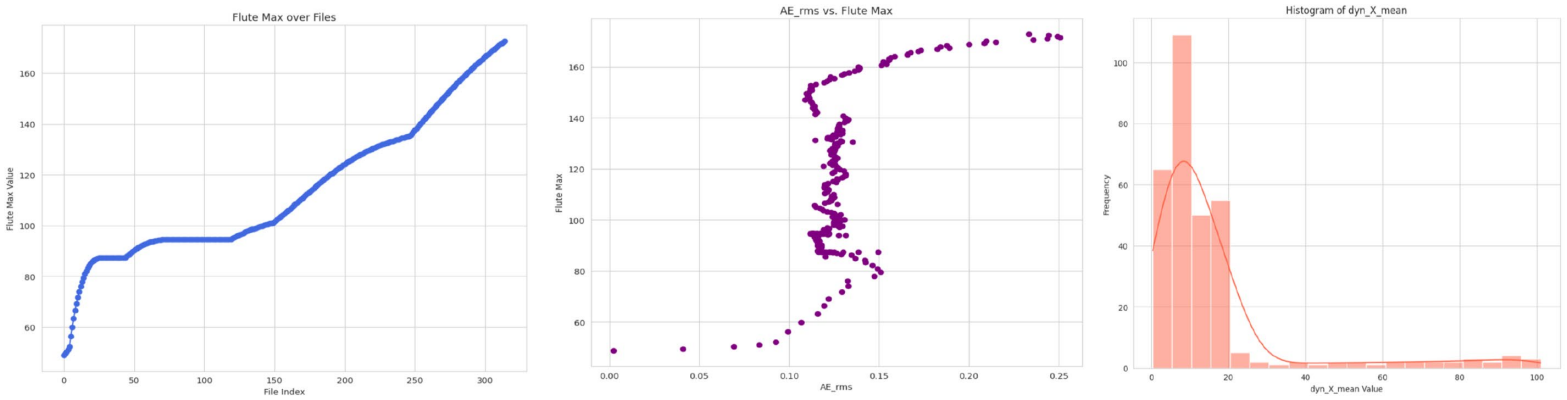
EDA visualization.

Additionally, to better understand the data and the progression of flute degradation after each cut, the wear files provided in the dataset were analyzed. A function was developed to traverse each cutter machine’s wear files and identify the maximum wear difference between sequential cuts. This information is crucial and will be utilized in the prediction process of the reinforcement learning agent. The maximum wear difference recorded was used as a hyperparameter in every MDP model. Further, an EDA was conducted to uncover patterns and relationships within the dataset. Figure 3 depicts some indicative visualizations.

Using the Gymnasium (formerly OpenAI Gym) library, we designed a custom environment for solving the RL optimization problem. It simulates a system where an agent makes predictions about the wear on certain components (referred to as “flutes”) in a manufacturing process.

The environments simulate the CNC machine’s wear prediction problem. The agent’s objective is to accurately predict the wear on three specific components (flutes) based on curated historical sensor data, with rewards and penalties assigned based on prediction accuracy.

The environments created for the training of the RL algorithm in this implementation are four. They are split into two major categories based on the Prediction Model and the Reward Calculation. All the environments work similarly but differ in the two major categories mentioned. The similar features are the State Space, Action Space and the Initial State Distribution:

The State Space is completely represented by the observation space (fully observable environment) and consists of 42 time-domain features derived from sensor data (7 sensors with 6 time domain features each), offering a detailed manufactured snapshot of the machine’s condition after each cut. All the data is derived from the dataframe that we provide to the environment class as an argument, which is the processed dataset we have created after completing the feature extraction.

Observations are constructed from the current step’s sensor data. An observation is part of a row of the dataframe (df) which is the PHM dataset we have processed. For each step in every episode, meaning for each cut we examine, we pick from the data frame all the time domain features corresponding to the cut (the current step). This observation will be used to determine the action by the policy in the current step.

The Action space is a continuous space with three dimensions, each ranging from 0 to 1. These actions represent the agent’s predictions for the additional wear on three flutes of the CNC machine. The prediction of the additional wear of each flute will be used to calculate the overall wear.

The Initial state is set up from the first observation of the machine coupled with some noise, initializing the environment with the machine wear values.

The Reward function evaluates the accuracy of the agent’s predictions. The closer the predictions are to the actual wear values, the higher the reward. The reward is calculated using the Score Function (Li, 2021) described below:


$$\text{Score Function}=\{\begin{array}{c} 1-e^{\left(-\frac{\delta }{10}\right)},\delta <0\\ 1-e^{\left(-\frac{\delta }{4.5}\right)},\delta >0. \end{array}$$

Where *δ* represents the difference between the predicted maximum wear and the actual maximum wear:


δ=Wear Prediction Value−Wear Actual Value

The score function is the negation of the original score function used in the 2010 PHM Data Challenge competition. The reason why the negation version of the function is used is that the agent wants to maximize its reward and the original version is a minimization function. It is designed to provide feedback to the agent based on the accuracy of its predictions, specifically focusing on penalizing overestimations more severely than underestimations. This design ensures that the agent learns to avoid predicting excessively low wear values, which can be more costly and disruptive. The use of exponential functions provides a smooth gradient for learning, allowing the agent to adjust its predictions incrementally and effectively.

Also, in order to remain consistent with the scoring methodology used in the 2010 PHM Data Challenge, as described in (Chen, 2011) we also implemented the original score function. This function is used solely as an evaluation metric to assess the performance of the models we develop, and it is not used as a training metric by the policy. We refer to this metric as the PHM Score, and we will use it to compare the performance of our model both internally and against other types of predictors, such as a dummy baseline model and supervised machine-learning methods, including logistic regression.

The environment resets to its starting state at the beginning of each episode, ensuring a consistent baseline for the agent’s learning process. The step function executes one-time step in the environment, updating the state, calculating the reward, and determining whether the episode should terminate or truncate.

The environment terminates when the current step exceeds the maximum number of steps (MAX_STEPS). In all the cut files provided by the PHM dataset, the maximum number of steps is 315, so the maximum episode length is 314.

The environment can also be truncated if the episode reward is smaller than −200 million. This is done to speed up the training process by cutting short poorly performing training episodes, allowing the agent to focus on more productive actions. Models that fail that consistently get truncated and never reach the maximum episode length are flagged are marked as “Did Not Finish” (DNF).

The differences between the four environments are the Prediction Method (Corrective, Non-Corrective) and Reward Calculation (Delay, No-Delay). Each combination of these categories defines a distinct environment within the MDP framework.

The “Corrective Prediction” is an approach where the agent’s predictions are corrected based on the actual wear values observed. This means that the agent adjusts its predictions by considering the current actual wear, providing a more accurate and real-time adjustment to the state of the environment.

The “Non-Corrective Prediction” involves updating the predictions incrementally based on previous predictions without immediate correction based on actual wear values. It relies on the agent’s previous predictions to inform future predictions.

Under the “No Delay” method the reward is calculated directly based on the difference between predicted and actual wear values, without any scaling factor related to the current step. This method focuses on immediate accuracy without considering the long-term impact of actions.

In contrast the “With Delay” method adjusts the reward using a “delay modifier,” which scales the reward based on the current step within the episode. This method aims to emphasize actions that provide long-term benefits by rewarding actions that have a positive effect later in the episode.

Combining the two categories, the four distinct environments are described in Table 4.

**Table 4:** The four distinct environments.

Environments	
Corrective with Delay	The agent’s predictions are corrected based on actual wear values, and the rewards are adjusted by a delay modifier to emphasize long-term benefits.
Corrective No Delay	The agent’s predictions are corrected based on actual wear values, and the rewards are calculated directly based on immediate prediction accuracy.
Non-Corrective with Delay	The agent’s predictions are incrementally updated based on previous predictions, and the rewards are adjusted by a delay modifier to emphasize long-term benefits.
Non-Corrective No Delay	The agent’s predictions are incrementally updated based on previous predictions, and the rewards are calculated directly based on immediate prediction accuracy.

### 4.3 Experimental results of RL model training and health state prediction

In this Section, we present the experimental results from the RL model training process. The framework produces a wear estimate at every step of an episode comprising 315 sequential identical cuts. Although the environments differ in terms of prediction method and reward calculation, they all aim to guide the agent toward accurately modeling the wear evolution throughout the cutting sequence. The score function associated with each environment is used as the primary performance metric and quantifies the alignment between the agent’s predictions and the true wear behavior. The results are derived from the four algorithms (PPO, SAC, DDPG, A2C) applied within the four different environments (Corrective-with Delay, Corrective-No Delay, Non-Corrective-with Delay, Non-Corrective-No Delay), thus having 16 distinct models. All models use the default hyper parameters provided by the Stable Baselines library. The training was conducted on the processed dataset of cutter 1 (train set). For each Prediction Method (i.e., Non-Corrective, Corrective), we demonstrate and compare the results for each RL algorithm for the two methods of Reward Calculation (No Delay, With Delay). For the Non-Corrective prediction method (Section 4.3.1), for each algorithm, we present the results of Mean Episode Length, Mean Episode Reward, and PHM Score per timestep in a graph. For the Corrective prediction method (Section 4.3.2), for each algorithm, we present the results of Mean Episode Reward, and PHM Score per timestep.

An increase in the numerical value of the mean episode length per timestep graph indicates that the agent is learning to act in a way that allows episodes to continue for longer without being truncated. In our environment, each cutting-file episode has a maximum possible length of 314 steps. Therefore, when the mean episode length reaches 314, it signifies that the agent successfully predicts the tool wear for all cuts in every episode and no longer encounters early truncation. The speed of increase in this curve reflects the rate of effective learning. A steeper rise means that the RL algorithm requires fewer timesteps to reach a level of performance where it can complete full episodes. Although a rapid learning rate does not necessarily imply that the resulting model is the best in overall performance, it can be advantageous in scenarios where fast adaptation is important, such as real-time applications and learning.

Mean Episode Score and PHM Score per Timestep are performance metrics. In the case of mean episode score the higher the score the better, since the agent tries to maximize it. The opposite is true for the Phm score which follows the logic of the PHM Data Challenge. The speed of increase in the Mean Episode Reward per Timestep graph represents how quickly the agent is improving its decision-making policy with respect to the reward function defined in the environment. A faster rise indicates that the agent is rapidly learning which actions lead to higher cumulative reward. Although rapid reward improvement suggests efficient learning, it does not necessarily imply the best final performance; some models may learn more slowly but ultimately achieve higher stability or better asymptotic results. In contrast, for the PHM Score, the interpretation depends on the direction of improvement. Since lower PHM Scores correspond to better performance (following the PHM Data Challenge scoring framework), a fast decrease in this metric reflects faster learning. A rapid decline means that the agent quickly reduces prediction errors associated with wear estimation.

The training process begins by creating an instance of the simulated environment using the preprocessed dataset. Each environment allows the RL agent to interact with it and learn to predict the wear of the machine. Then, the environment is initialized and sets up a logging mechanism to track the progress and performance of the models. A key aspect of this setup is defining the number of timesteps for training and creating a directory structure to store the training logs and model files. A callback function was implemented to log the score during training and to monitor the performance of the agent at various steps. To ensure efficient and organized training, a function was developed to manage the creation and naming of model files. This function also can load previously trained models, enabling the continuation of training from the last saved state. During the training loop, models are periodically saved, and their performance is evaluated to ensure they are learning effectively. The training process is executed in iterations, with each iteration involving a specified number of timesteps. For each episode, the model predicts the next action based on the current observation. After each iteration, the models are evaluated, and their performance metrics are logged. The hyper parameters used for each RL algorithm are shown in Table 5.

**Table 5:** The hyper parameters used for each RL algorithm.

Hyper Parameters for all RL Algorithms Used	
PPO	policy = MlpPolicy, learning_rate = 0.0003, n_steps = 2048, batch_size = 64, n_epochs = 10, gamma = 0.99, gae_lambda = 0.95, clip_range = 0.2, clip_range_vf = None, normalize_advantage = True, ent_coef = 0.0, vf_coef = 0.5, max_grad_norm = 0.5, use_sde = False, sde_sample_freq = 1, rollout_buffer_class = None, rollout_buffer_kwargs = None, target_kl = None, stats_window_size = 100, tensorboard_log = logdir, policy_kwargs = None, verbose = 1, seed = None, device = ‘auto’, _init_setup_model = True
A2C	policy = MlpPolicy, learning_rate = 0.0007, n_steps = 5, gamma = 0.99, gae_lambda = 1.0, ent_coef = 0.0, vf_coef = 0.5, max_grad_norm = 0.5, rms_prop_eps = 1e-05, use_rms_prop = True, use_sde = False, sde_sample_freq = −1, rollout_buffer_class = None, rollout_buffer_kwargs = None, normalize_advantage = False, stats_window_size = 100, tensorboard_log = None, policy_kwargs = None, verbose = 0, seed = None, device = ‘auto’, _init_setup_model = True
DDPG	policy = MlpPolicy, learning_rate = 0.001, buffer_size = 1,000,000, learning_starts = 100, batch_size = 256, tau = 0.005, gamma = 0.99, train_freq = 1, gradient_steps = 1, action_noise = None, replay_buffer_class = None, replay_buffer_kwargs = None, optimize_memory_usage = False, n_steps = 1, tensorboard_log = None, policy_kwargs = None, verbose = 0, seed = None, device = ‘auto’, _init_setup_model = True
SAC	policy = MlpPolicy, learning_rate = 0.0003, buffer_size = 1,000,000, learning_starts = 100, batch_size = 256, tau = 0.005, gamma = 0.99, train_freq = 1, gradient_steps = 1, action_noise = None, replay_buffer_class = None, replay_buffer_kwargs = None, optimize_memory_usage = False, n_steps = 1, ent_coef = ‘auto’, target_update_interval = 1, target_entropy = ‘auto’, use_sde = False, sde_sample_freq = −1, use_sde_at_warmup = False, stats_window_size = 100, tensorboard_log = None, policy_kwargs = None, verbose = 0, seed = None, device = ‘auto’, _init_setup_model = True

The experiments were conducted on a personal workstation, specifically an Asus TUF DASH F15 equipped with an Intel 12th Gen Core i5-12450H processor, 16 GB DDR5 RAM, and an NVIDIA GeForce RTX 3050 GPU. This configuration provides sufficient computational resources for training and evaluating the approach’s RL agents, while remaining relatively entry-level compared to hardware commonly available in research or industrial settings. This makes the proposed approach computationally feasible even on modest hardware, such as a standard high-performance laptop, and highlights its suitability for real-time industrial applications where access to server-grade resources may be limited.

#### 4.3.1 Non-corrective prediction

##### 4.3.1.1 The PPO algorithm

**Figure 4:**
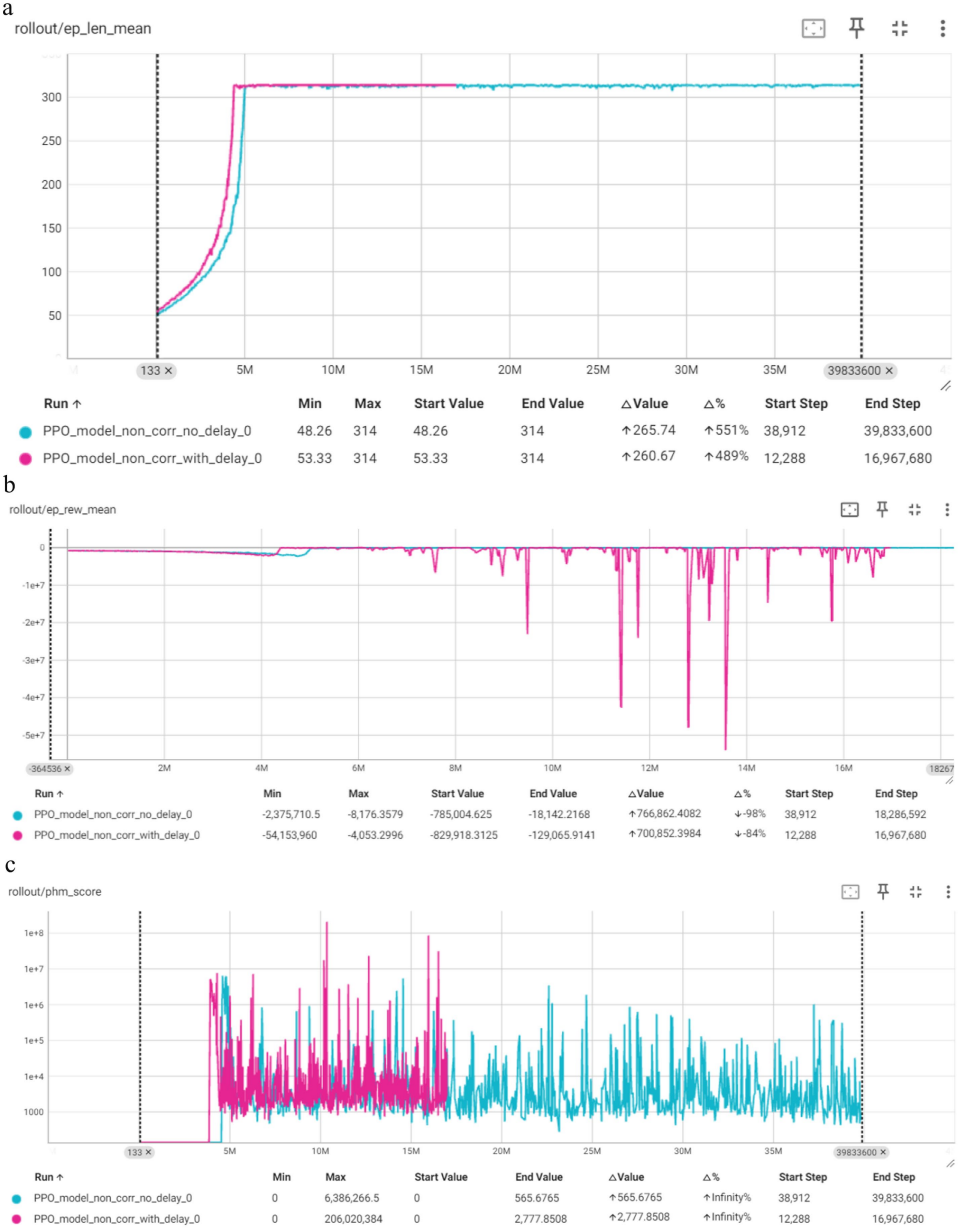
Comparison of PPO in terms of: **(a)** Mean episode length (Y-axis) per timestep (X-axis); **(b)** mean episode reward (Y-axis) per timestep (X-axis); **(c)** PHM score (Y-axis) per timestep (X-axis).

Figure 4 indicates that the model “with delay” reaches episode length of 314 faster than the “no delay” model, suggesting more efficient learning early on. Despite this, both models ultimately achieve the same final performance, which is reasonable. The model “with delay” exhibits significantly larger fluctuations, including deep spikes. Over time, both models improve. The model “with delay” shows higher initial PHM scores and greater fluctuations compared to the model “no delay.” Over time, both models reduce the PHM scores. We observe that delay correction may accelerate learning initially, it results in higher variability and less optimal final performance in minimizing PHM scores.

##### 4.3.1.2 The SAC algorithm

**Figure 5:**
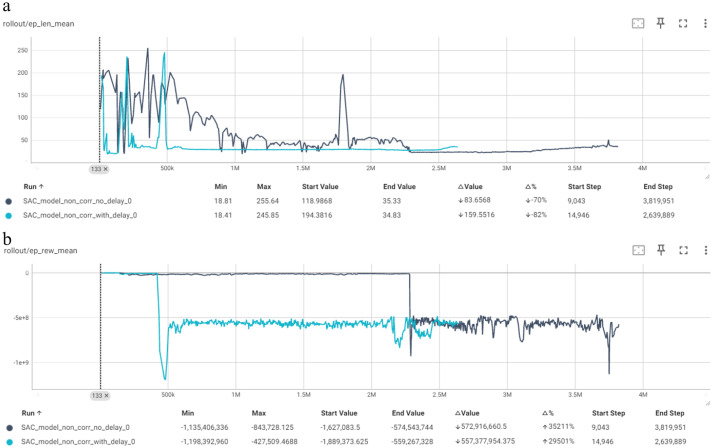
Comparison of SAC in terms of: **(a)** Mean episode length (Y-axis) per timestep (X-axis); **(b)** mean episode reward (Y-axis) per timestep (X-axis).

Neither the “no delay” model not the ‘with delay’ model reached the 314-cut mark so the PHM score cannot be examined. The models were constantly truncated due to poor performance. A shown in Figure 5, the comparison of SAC shows that both exhibit a downward trend in mean episode length and rewards and both models have not reached the max episode length of 314. The “no delay” model starts at 118.98 and ends at 35.33, while the ‘with delay’ model starts at 194.38 and ends at 34.83, indicating that both struggle to maintain longer episodes. Both models have highly negative rewards, highlighting their poor performance.

##### 4.3.1.3 The DDPG algorithm

**Figure 6:**
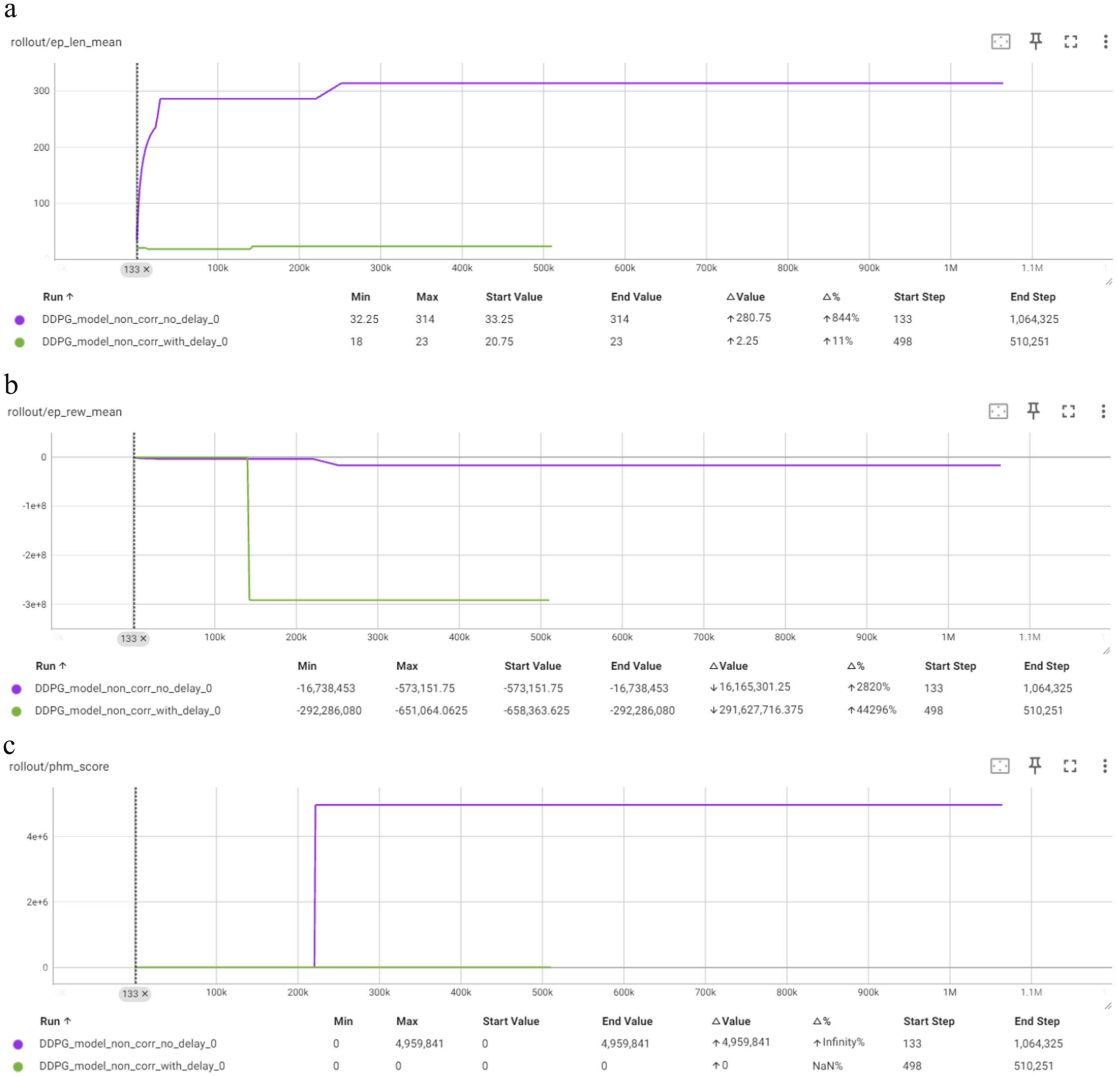
Comparison of DDPG in terms of: **(a)** Mean episode length (Y-axis) per timestep (X-axis); **(b)** Mean episode reward (Y-axis) per timestep (X-axis); **(c)** PHM score (Y-axis) per timestep (X-axis).

As shown in Figure 6, the comparison of DDPG models reveals significant differences in performance. The “no delay” model quickly achieves and maintains the maximum mean episode length of 314, while the ‘with delay’ model shows only a minor increase from 20.75 to 23. Overall, the “no delay” model is better, but it fails to optimize the reward metrics. The deterministic nature of the algorithm prohibits the improvement in terms of reward.

##### 4.3.1.4 The A2C algorithm

**Figure 7:**
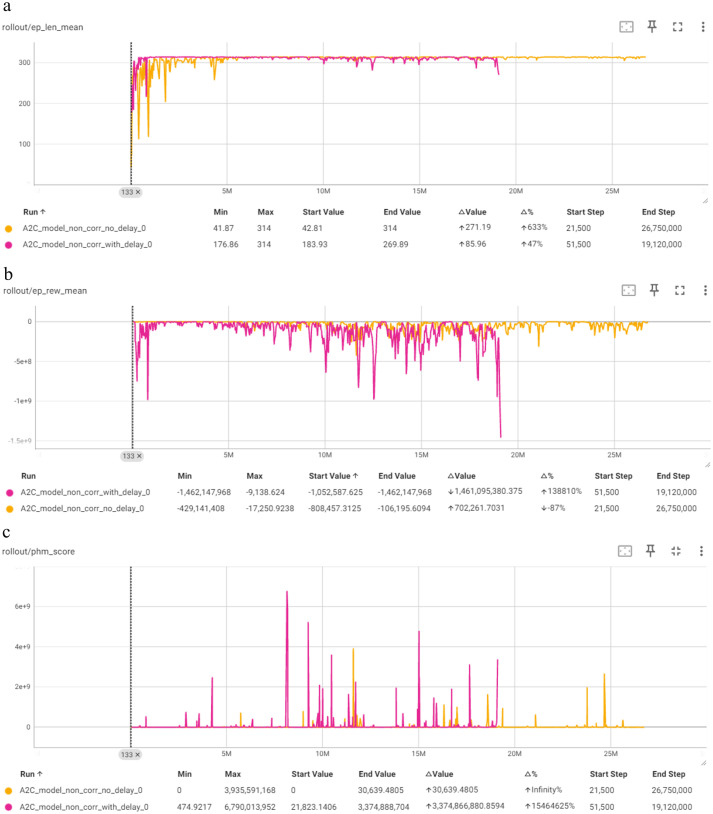
Comparison of A2C in terms of: **(a)** Mean episode length (Y-axis) per timestep (X-axis); **(b)** mean episode reward (Y-axis) per timestep (X-axis); **(c)** PHM score (Y-axis) per timestep (X-axis).

As shown in Figure 7, the “no delay” model initially shows significant variance in mean episode length but stabilizes at the maximum value of 314, indicating that it eventually learns to maximize episode duration although slower. Its mean episode reward graph, however, reveals large negative rewards, reflecting high variance and instability throughout the training process. Also, the corresponding PHM score graph shows substantial variability, indicating that the model often deviates from optimal behavior. On the contrary, the ‘with delay model’, while showing similar initial fluctuations, converges more steadily and faster. The PHM score also indicates more consistent performance with fewer extreme values compared to the “no delay” model.

#### 4.3.2 Corrective prediction

##### 4.3.2.1 The PPO algorithm

**Figure 8:**
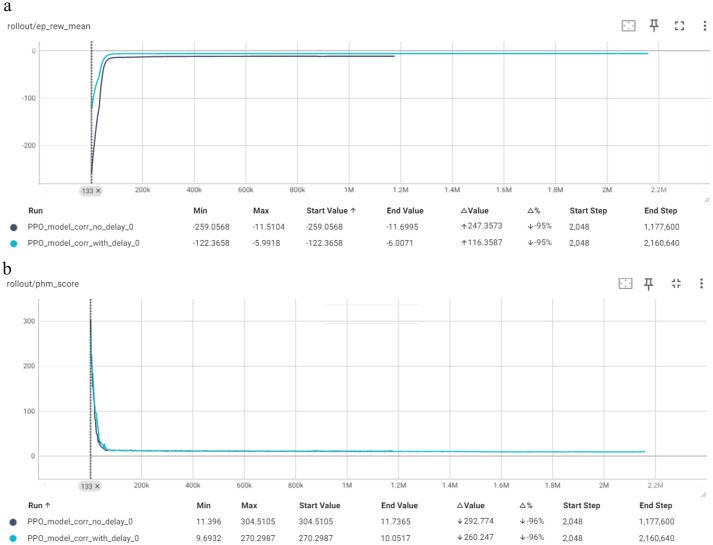
Comparison of PPO in terms of: **(a)** Mean episode reward (Y-axis) per timestep (X-axis); **(b)** PHM score (Y-axis) per timestep (X-axis).

Figure 8 depicts the comparison of the “no delay” and “with delay” models for the PPO algorithm in terms of Mean Episode Reward per Timestep and PHM Score per Timestep. The “no delay” model converges quickly into the max episode length, with a significant increase observed within the first 100,000 steps. The mean episode reward increases by 95% from its initial value, indicating successful training. The PHM Score also follows a similar trend, rapidly decreasing to a near-zero value within the same timeframe, which aligns with the reduction in variance and stabilization of the policy. This behavior highlights the model’s efficiency in reaching optimal performance quickly and reliably. The “with delay” model demonstrates a rapid increase in mean episode reward, reaching stability early and maintaining it throughout the training period. The reward improves from −122.3 to −6.0, representing a positive change of +116.3 (95%). The PHM score also shows a significant reduction from its peak, settling at around 10.05 with a decrease of 260 (96%). Both models exhibit efficient learning, quickly stabilizing their rewards and PHM scores, indicating successful convergence and effective training in the corrective environment. The only slight difference is that the “with delay” model is slightly faster in converging.

##### 4.3.2.2 The SAC algorithm

**Figure 9:**
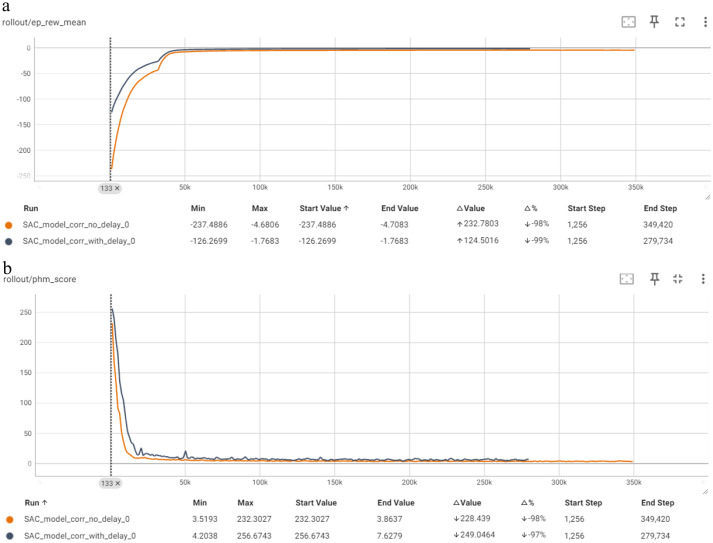
Comparison of SAC in terms of: **(a)** Mean episode reward (Y-axis) per timestep (X-axis); **(b)** PHM score (Y-axis) per timestep (X-axis).

Figure 9 depicts the comparison of the “no delay” and “with delay” models for the SAC algorithm in terms of Mean Episode Reward per Timestep and PHM Score per Timestep. The “no delay” model demonstrates a rapid convergence in terms of mean episode reward, with a significant increase up to around 237.4. The reward stabilizes quickly, indicating efficient learning. The PHM score also shows a rapid decrease. Overall, this model shows effective and stable performance throughout the training period. The “with delay” model shows a faster increase in the mean episode length per timestep, but a slower and more gradual increase in mean episode reward, peaking at around −1.76 (perfect score). The learning process appears to be slowed by the “with delay” model, has slightly less efficient learning and lower overall rewards compared to the ‘no delay’ model. The PHM score similarly decreases but stabilizes at a higher level of uncertainty than the “no delay” model, indicating less efficient convergence.

##### 4.3.2.3 The DDPG algorithm

**Figure 10:**
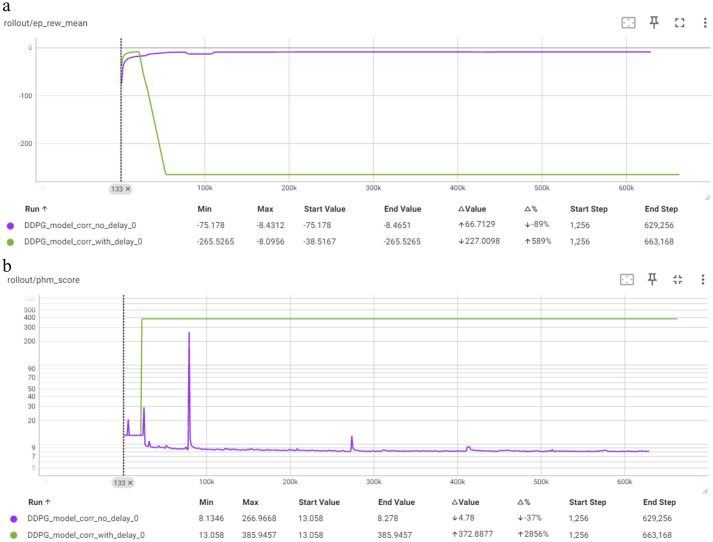
Comparison of DDPG in terms of: **(a)** mean episode reward (Y-axis) per timestep (X-axis); **(b)** PHM score (Y-axis) per timestep (X-axis).

Figure 10 depicts the comparison of the “no delay” and “with delay” models for the DDPG algorithm in terms of Mean Episode Reward per Timestep and PHM Score per Timestep. The “no delay” model exhibits a significant increase in mean episode reward, improving from −75.1 to −8.46, representing an 89% improvement. However, the PHM Score shows minimal improvement, with a change of −37%. This model manages to perform better over time but does not reach the optimal performance level of other models, as indicated by the relatively small decrease of PHM Score. The “with delay” model demonstrates a severe drop in performance. The mean episode reward heavily decreases, and the PHM Score increases dramatically. This model appears to be stuck in a bad policy, leading to poor performance.

Comparing the two DDPG corrective models, the” no delay” model shows better performance with a higher mean episode reward improvement and a decent PHM Score. The” with delay” model indicates poor performance overall with significant drops in mean episode rewards and increased actor loss variability. The comparison highlights that the discounted rewards as the” with delay” model is not good in a DDPG algorithm.

##### 4.3.2.4 The A2C algorithm

**Figure 11:**
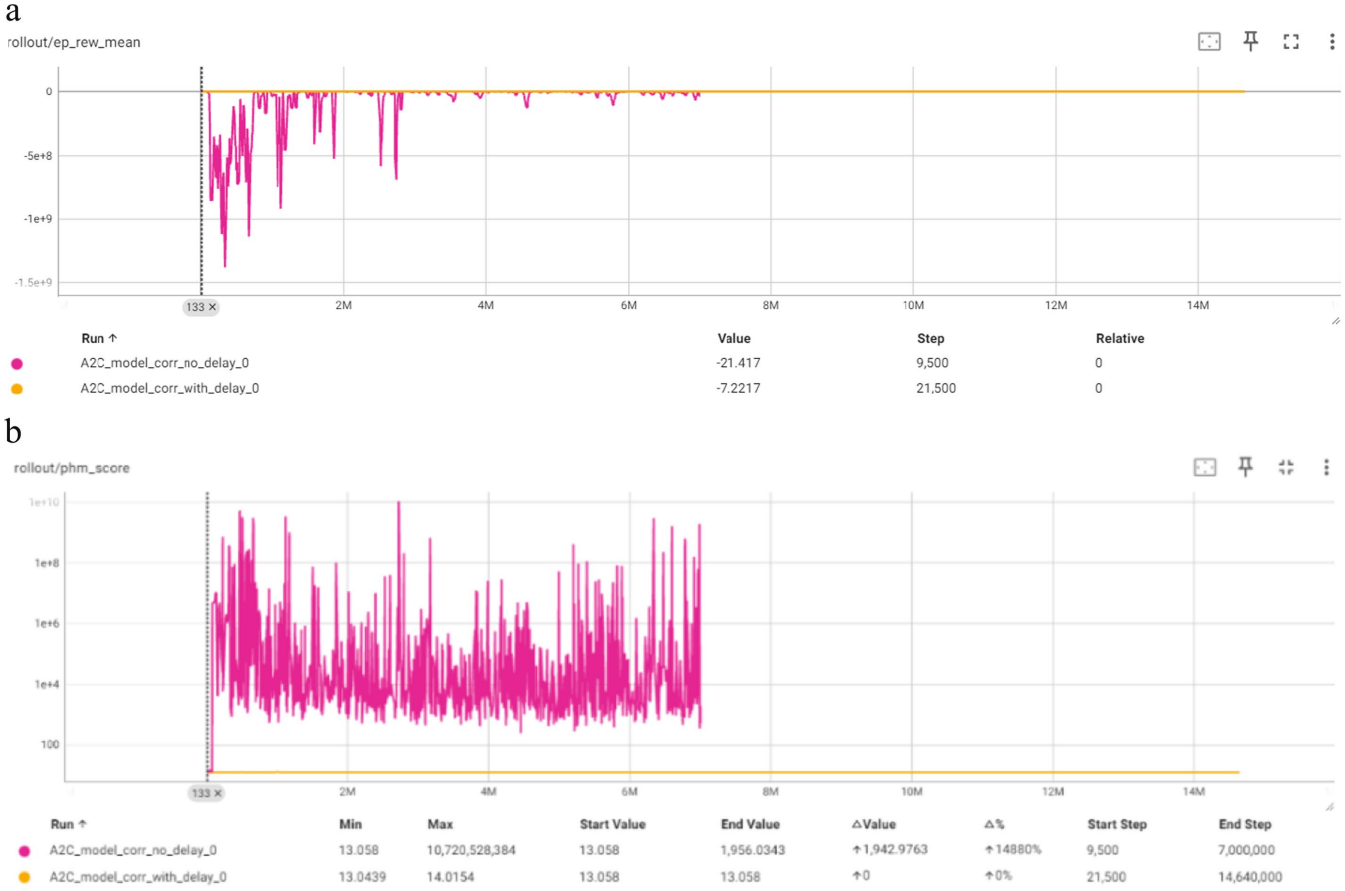
Comparison of A2C corrective “with delay” and “no delay” models for: **(a)** Mean episode reward (Y-axis) per timestep (X-axis); **(b)** PHM score (log scale) (Y-axis) per timestep (X-axis).

Figure 11 depicts the comparison of the “no delay” and “with delay” models for the A2C algorithm in terms of Mean Episode Reward per Timestep and PHM Score per Timestep. The “no delay” model is the only Corrective model that does not reach the max episode length on the first few timesteps. It shows significant fluctuations in the mean episode length initially, stabilizing after around three million timesteps. The mean episode reward graph indicates substantial variations in reward values and the PHM score also demonstrates considerable variability, reflecting a challenging learning environment. The “with delay” model shows a consistent mean episode length near the maximum limit, with minor early fluctuations. The mean episode reward graph indicates a stable performance with minor improvements over time. The PHM score graph remains flat, indicating that the model did not significantly improve in this metric, but it still has a good score.

### 4.4 Overview of the training results

**Figure 12:**
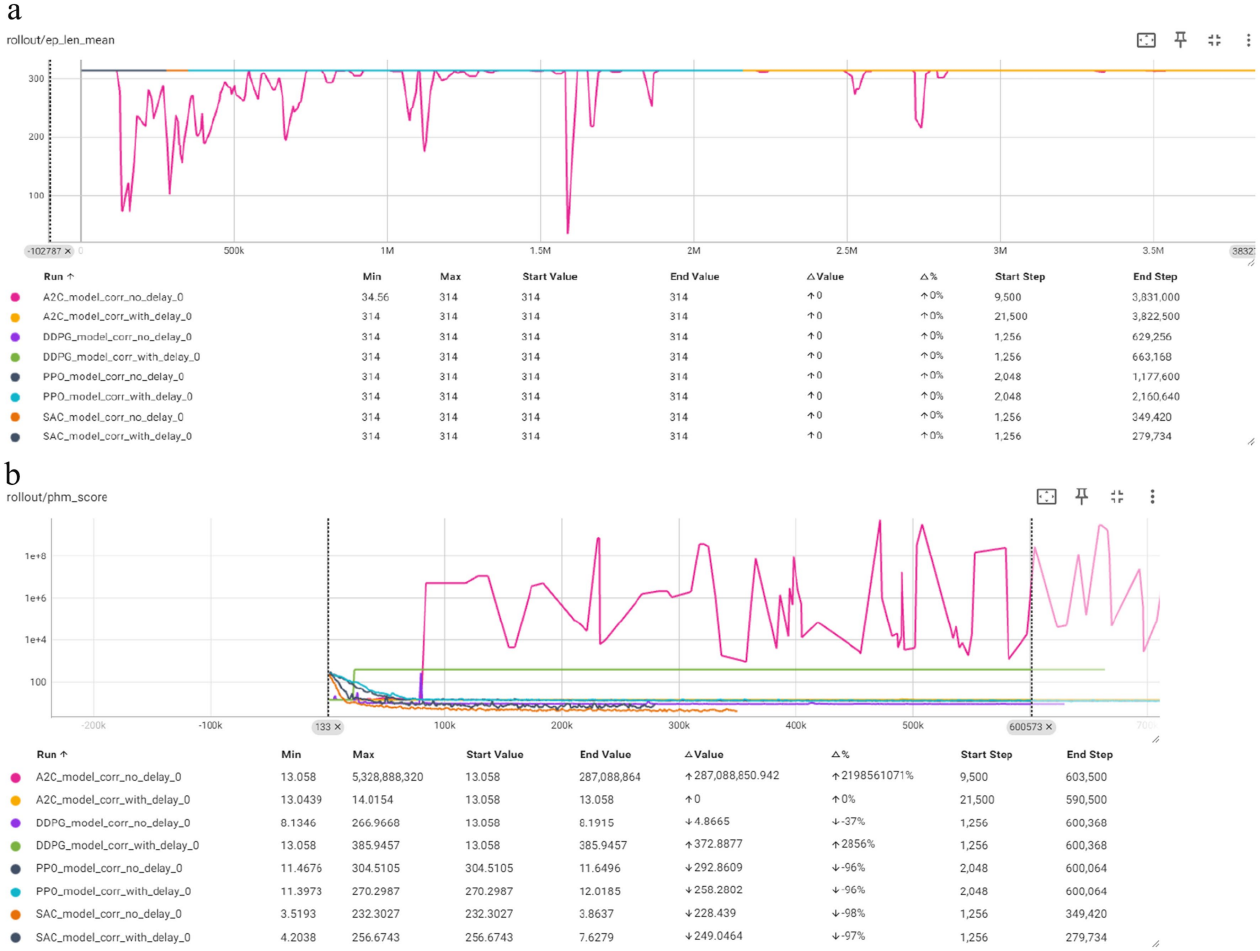
Overview of the training results for the corrective prediction: **(a)** mean episode reward (Y-axis) per timestep (X-axis); **(b)** PHM score (Y-axis) per timestep (X-axis).

In this Section, we present an overview of the training results, summarizing the RL models performance for equipment health state prediction. Figure 12 depicts the overview of the results from the four RL algorithms training embedded in the “no delay” and “with delay” models for the Corrective Prediction Method (8 models per Category) as well as the overview of the results from the four RL algorithms embedded in the “no delay” and “with delay” models for the Non-Corrective Prediction Method.

The graph showcasing the mean episode reward per timestep clearly displays the A2C “no delay” model attempt of big exploration. While most models eventually stabilize, they show varying levels of performance improvement. The A2C model “no delay” experiences significant negative rewards, reflecting its difficulty in learning initially. Over time, other models, particularly those “with delay” correction, show more consistent improvements in rewards. With DDPG showing the best performance early on and PPO showing consistent improvement and surpasses other models.

Once again in the PHM Score per timestep graph, A2C “no delay” fluctuates greatly, while A2C “with delay” remains stable. The DDPG “no delay” model initially fluctuates but then remains steady, and DDPG “with delay” shows increased exploitation remaining almost constant after the initial exploration. Both PPO models consistently maintain low, stable scores, indicating strong performance. SAC models also perform well with stable, low scores. Overall, PPO and SAC are the most robust, “with delay” correction improving stability for A2C and DDPG.

**Figure 13:**
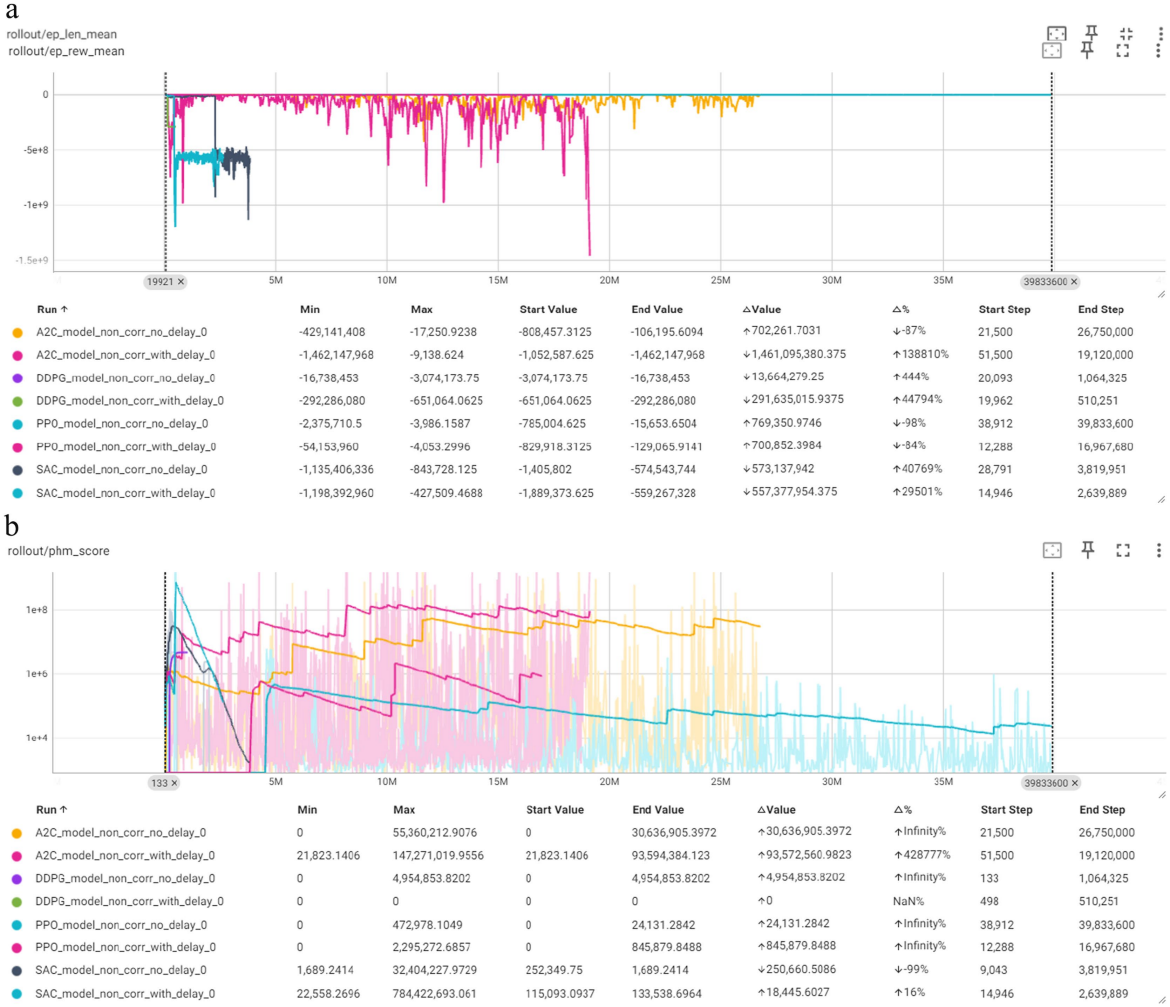
Overview of the training results for the non-corrective prediction: **(a)** mean episode reward (Y-axis) per timestep (X-axis); **(b)** PHM score (Y-axis) per timestep (X-axis).

Figure 13 provides a comparison of the performance of the RL algorithms (A2C, DDPG, PPO, SAC) “with delay” and “no delay” correction in terms of mean episode length over training steps. PPO stands out for its consistent and strong performance. Both PPO models, “with delay” and “no delay” correction, demonstrate significant improvements and stable learning trajectories, stabilizing around the maximum episode length of 314. This indicates that PPO is highly effective for the task at hand, with or without the additional reward scaling introduced by the “with delay” correction.

For A2C, the models show notable improvements in episode length, with the version” no delay” correction achieving a slightly better episode length of 311.87 compared to the version “with delay” correction (308.91). The “with delay” correction helps the A2C model stabilize more quickly, suggesting that while it aids in the learning process, the final performance is slightly better without it.

DDPG models exhibit more alternation in performance. The DDPG model” no delay” correction reaches the max episode length of 314, indicating effective learning, though it has minimal fluctuations during training. In contrast, the DDPG model “with delay” correction struggles, showing minimal improvement and achieving only a slight increase from 20.75 to 23, suggesting that “with delay” correction does not benefit DDPG in this context.

SAC models perform poorly compared to the other algorithms. Both SAC models, “with delay” and “no delay” correction, show a significant decline in episode length over time. The SAC model” no delay” correction starts at 118.98 and drops to 22.51, while the SAC model “with delay” correction starts at 84.08 and drops to 28.69. This suggests that SAC struggles with this particular task and the “with delay” correction aids in the decline in performance.

### 4.5 Discussion of evaluation results

These are the results from evaluating the 16 RL models on the test set, which consists of cutters 4 and 6 from the PHM dataset. Table 6 presents the aggregated performance metrics for the models in the Corrective environments, while Table 7 presents the corresponding aggregated metrics for the Non-Corrective environments.

**Table 6:** Performance of RL algorithms in corrective environments.

Corrective prediction							
RL algorithm	Environment and dataset (cutters)		Max reward		Min PHM score		Training time and total timesteps
			Value	Timestep	Value	Timestep	
PPO	No Delay	c4	−17.21	40,000	16.99	40,000	3 h 1,117,000 steps
		c6	−16.21	70,000	18	70,000	
	With Delay	c4	−8.98	20,000	15.73	20,000	2 h 2,160,000 steps
		c6	−8.23	1,460,000	17.85	670,000	
SAC	No Delay	c4	−37.37	90,000	45.76	90,000	6.2 h 349,420 steps
		c6	−14.8	30,000	16.36	30,000	
	With Delay	c4	−10.94	20,000	23.07	20,000	5.3 h 279,374 steps
		c6	−8.21	110,000	16.22	110,000	
DDPG	No Delay	c4	−18.84	20,000	19.32	20,000	12.67 h 629,256 steps
		c6	−17.04	250,000	18.55	520,000	
	With Delay	c4	−10.75	20,000	19.36	20,000	11.5 h 452,160 steps
		c6	−8.83	20,000	18.12	20,000	
A2C	No Delay	c4	−17.84	700,000	18.33	700,000	13.7 h 19,120,000 steps
		c6	−16.33	7,000,000	18.12	7,000,000	
	With Delay	c4	−10.75	14,640,000	19.36	14,640,000	17.2 h 14,640,000 steps
		c6	−8.83	14,640,000	18.12	14,640,000	

**Table 7:** Performance of RL algorithms in non-corrective environment.

Non-Corrective Prediction							
RL algorithm	Environment and dataset (Cutters)		Max reward		Min PHM score		Training time and total timesteps
			Value	Timestep	Value	Timestep	
PPO	No Delay	c4	−25,255,715	38,730,000	35,497,029	38,730,000	17 h 39,833,600 steps
		c6	−163,762,286	20,000	233,645,404	20,000	
	With Delay	c4	−544,135,308	20,000	726,430,945	20,000	19,2 h 16,967,680 steps
		c6	−583,253,362	20,000	876,304,975	20,000	
SAC	No Delay	c4	−643	400,000	752	390,000	43.3 h 3,819,951 steps
		c6	−5,679	410,000	3,140	460,000	
	With Delay	c4	−23,455	200,000	11,418	200,000	32.1 h 2,639,889 steps
		c6	−15,048	470,000	7,712	190,000	
DDPG	No Delay	c4	−563,092,188	1,050,000	735,266,738	1,050,000	19.5 h 1,064,325 steps
		c6	−637,838,860	1,050,000	910,064,743	1,050,000	
	With Delay	c4	DNF	-	DNF	-	13.5 h 510,251 steps
		c6	DNF	-	DNF	-	
A2C	No Delay	c4	−50,834,200	30,000	3,469,607	30,000	26.1 h 26,750,000 steps
		c6	−28,469,419	30,000	39,536,971	30,000	
	With Delay	c4	−550,755,354	19,110,000	735,266,790	19,110,000	22.8 h 19,120,000 steps
		c6	−606,030,553	19,110,000	910,064,743	19,110,000	

The comparative evaluation of the four RL algorithms—PPO, SAC, A2C, and DDPG—demonstrates distinct performance characteristics across training efficiency, convergence behavior, adaptability to delay, and generalization across environments.

PPO consistently proves to be the most robust and reliable algorithm. It shows high and stable performance across all environments, with fast training times per timestep and efficient convergence. Its ease of implementation and low computational demand further reinforce its practical utility. PPO excels particularly in Corrective environments, demonstrating stable episode lengths and rewards across time. The “with delay” correction improves adaptability to long-term rewards, as evident from the improved performance in delayed environments, especially for dataset C6. However, saturation occurs early in training, suggesting overfitting risk if the process is not monitored. In Non-Corrective environments, PPO models—with and without delay—maintain strong performance and stable episode lengths near the maximum (314), with minimal sensitivity to reward delay correction.

SAC, although computationally more intensive and complex due to the need for multiple network structures (policy, Q-function, and value function), shows exceptional adaptability in Corrective environments. The algorithm converges the fastest and requires the fewest timesteps, with entropy-based exploration enabling it to find optimal policies quickly. However, despite its strong early-stage performance and robustness against overfitting in the Corrective setting, SAC underperforms in the Non-Corrective environment. The results show a steady decline in mean episode length over time for both SAC variants. Reward plots indicate a lack of consistent policy improvement, and Phm scores show high instability and spikes, reflecting reduced learning effectiveness. This contrast highlights that while SAC is powerful in structured environments, it struggles with more chaotic or loosely defined tasks, possibly due to exploration strategies that become less effective without clear corrective signals.

A2C shows consistent, though slower, improvement over time. In Corrective environments, especially without delay, the algorithm exhibits significant variance in reward and episode length, with notable negative scores early in training. With delay correction, A2C becomes more stable but still lags behind PPO and SAC in overall performance. Nevertheless, A2C is the only model across all cases where performance consistently improves over time rather than peaking early. In the Non-Corrective environment, A2C displays improved mean episode lengths, especially without delay correction (311.87 vs. 308.91), showing that delay correction stabilizes the model faster, though ultimate performance may be better without it. However, A2C still suffers from high reward variability and instability in Phm scores, suggesting limited generalization in noisy or unstable conditions.

DDPG is the most problematic among the four algorithms. Although theoretically suitable for continuous action spaces, its deterministic policy and poor exploration lead to consistently poor results. In all environments, DDPG converges to local optima early and fails to improve over time. Particularly in the Non-Corrective environment with delay, the model completely fails to reach the episode length of 314 and is terminated early due to extremely low rewards. Reward and Phm score plots confirm stagnant behavior with high negative values and flat trajectories, indicating a lack of meaningful learning. DDPG without delay performs marginally better, reaching maximum episode length in some cases, but still suffers from high Phm scores and erratic reward patterns.

Across all algorithms, it is noteworthy that best results are often achieved mid-training rather than at the end. This suggests potential overfitting as training continues—models begin to memorize training data patterns including noise, reducing generalization capacity. This trend is especially pronounced in SAC and PPO, both of which show strong early performance but limited improvement or even regression with prolonged training. Regarding delay correction, its impact varies significantly. While it aids A2C by stabilizing training and improves PPO’s long-term reward adaptation, it negatively affects DDPG, likely due to compounding its already poor exploration capabilities. For SAC, delay correction slightly mitigates performance decline but does not reverse the general trend of degradation in Non-Corrective environments.

To better interpret the evaluation results, a supervised ML baseline was incorporated for comparison with the RL algorithms. An XGBoost regressor was incorporated as a supervised learning baseline due to its strong performance in modeling nonlinear degradation patterns and its established effectiveness in predictive maintenance applications. The XGBoost Python library was used, and the model was implemented with all hyperparameters set to their default values, consistent with the approach taken for the RL models. A linear predictor was also implemented, incrementing the wear value by a fixed amount at each time step (e.g., +1 mm). This predictor exhibited substantially inferior performance, even when tested on different fixed increment values it had on average approximately 230% worse and was therefore excluded from the figures to maintain clarity.

**Figure 14:**
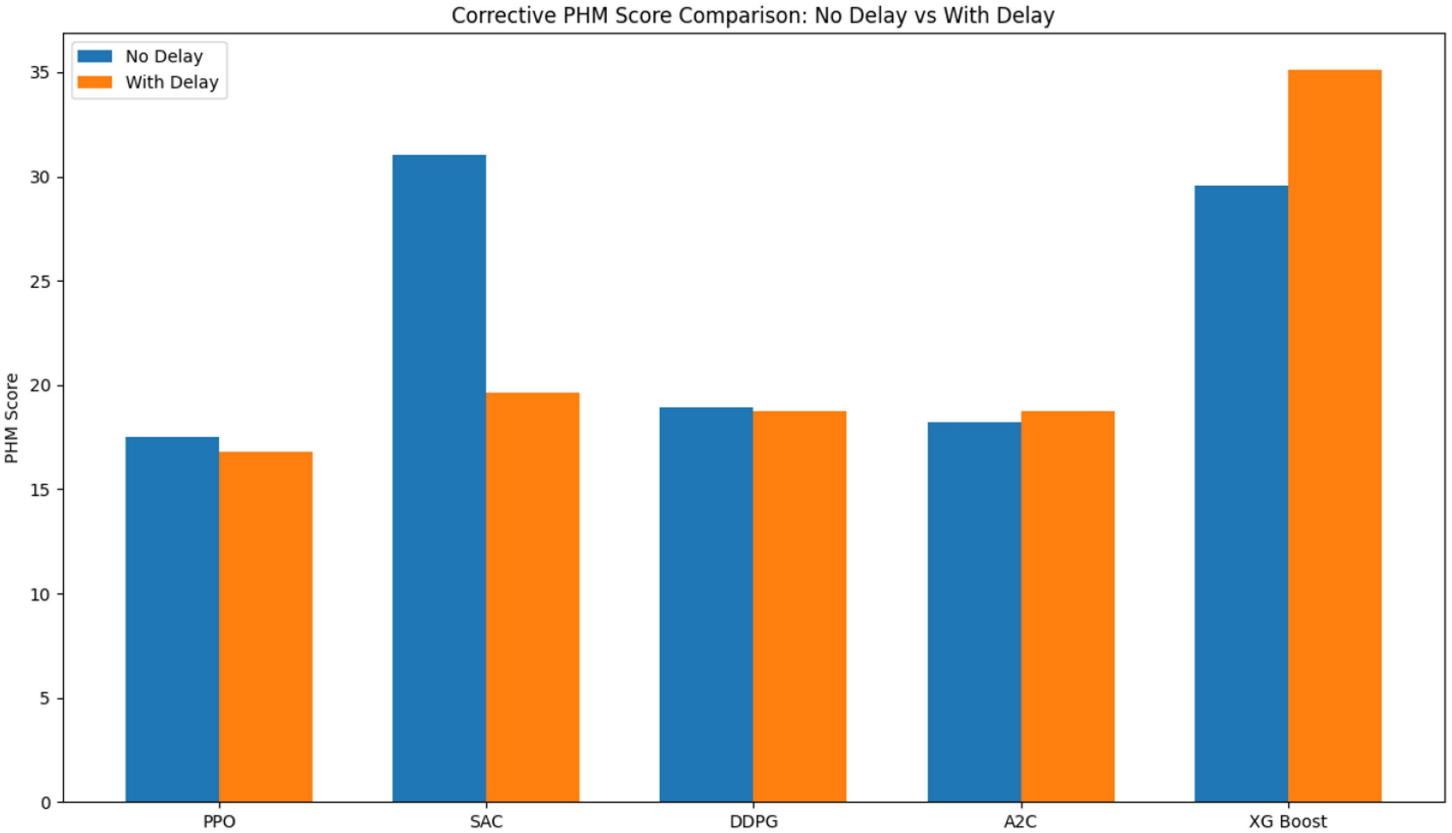
Corrective PHM score of each RL model, including both the with delay and no delay variants, across the two test cutters (cutter 4 and cutter 6).

**Figure 15:**
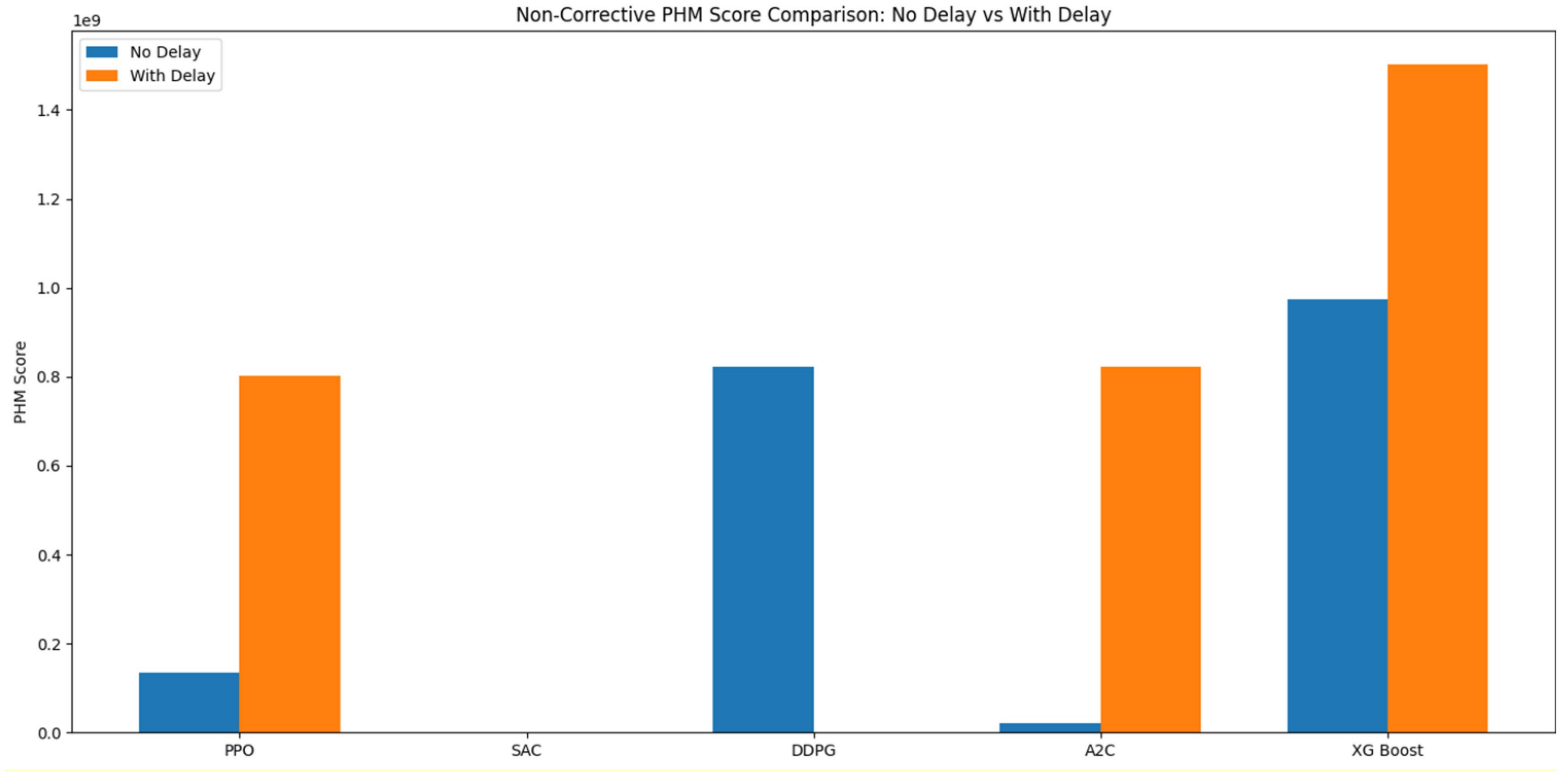
Non-corrective PHM score of each RL model, including both the with delay and no delay variants, across the two test cutters (Cutter 4 and Cutter 6).

Figures 14, 15 report the mean performance of each RL model, including both the With Delay and No Delay variants, across the two test cutters (Cutter 4 and Cutter 6). The XG Boost baseline is included for reference. In the Corrective Environment, we observe that the RL models significantly outperform the XG Boost baseline. This provides strong evidence that our structured RL approach is more effective for this task and highlights the advantages of learning decision policies rather than relying solely on supervised prediction. On the Non-Corrective Environment, the RL models again achieve superior performance, only those capable of reaching the maximum allowable number of steps within an episode without being designated as Did Not Finish (DNF). This outcome reflects the robustness of the RL approach, especially the RL Algorithms PPO and A2C and suggests that they provide a strong foundation for further methodological enhancement.

## 5 Conclusions and future work

This study demonstrated the potential of RL for equipment health state prediction within the context of predictive maintenance. By formulating the wear-estimation task as an MDP and evaluating four model-free RL algorithms (PPO, A2C, DDPG, and SAC) across corrective and non-corrective environments, we provided a systematic assessment of their learning behavior, convergence characteristics, and generalization performance on CNC machine data from the 2010 PHM Society Data Challenge.

The results highlight PPO as the most stable and computationally efficient method, achieving consistent convergence and strong generalization across all environments. SAC exhibited rapid and robust performance in structured corrective settings but struggled in non-corrective ones, indicating a sensitivity to environment design. A2C showed gradual, steady learning, making it suitable for applications requiring long-term stability. In contrast, DDPG consistently underperformed due to limited exploration and instability, especially in delayed-reward and unstructured scenarios. Overall, the findings confirm that RL can effectively capture the sequential and uncertain nature of machine degradation without relying on labeled failure data. They also emphasize the importance of aligning algorithm choice with environment characteristics—particularly regarding reward shaping and delay handling—to ensure reliable predictive performance.

Future work will extend this framework toward multi-agent RL for coordinated maintenance of multiple assets, as well as investigate adaptive reward mechanisms and online learning strategies for deployment in dynamic industrial settings.

## Data Availability

The original contributions presented in the study are included in the article/supplementary material, further inquiries can be directed to the corresponding author.
